# Identification of transcripts with short stuORFs as targets for DENR•MCTS1-dependent translation in human cells

**DOI:** 10.1038/s41598-017-03949-6

**Published:** 2017-06-16

**Authors:** Sibylle Schleich, Julieta M. Acevedo, Katharina Clemm von Hohenberg, Aurelio A. Teleman

**Affiliations:** German Cancer Research Center (DKFZ), 69120 Heidelberg, Germany

## Abstract

The non-canonical initiation factors DENR and MCTS1 have been linked to cancer and autism. We recently showed in Drosophila that DENR and MCTS1 regulate translation re-initiation on transcripts containing upstream Open Reading Frames (uORFs) with strong Kozak sequences (stuORFs). Due to the medical relevance of DENR and MCTS1, it is worthwhile identifying the transcripts in human cells that depend on DENR and MCTS1 for their translation. We show here that in humans, as in Drosophila, transcripts with short stuORFs require DENR and MCTS1 for their optimal expression. In contrast to Drosophila, however, the dependence on stuORF length in human cells is very strong, so that only transcripts with very short stuORFs coding for 1 amino acid are dependent on DENR and MCTS1. This identifies circa 100 genes as putative DENR and MCTS1 translational targets. These genes are enriched for neuronal genes and G protein-coupled receptors. The identification of DENR and MCTS1 target transcripts will serve as a basis for future studies aimed at understanding the mechanistic involvement of DENR and MCTS1 in cancer and autism.

## Introduction

DENR and MCTS1 are two non-canonical initiation factors that form a complex^1^. MCTS1 was identified by the Gartenhaus lab as an oncogene that is genomically amplified in T-cell leukemias^2^. Subsequent studies found that MCTS1 protein levels are also elevated in T-cell lymphoid cell lines, in non-Hodgkin lymphoma cell lines, and in 85% of primary diffuse large B-cell lymphomas^3^. MCTS1 has oncogenic properties, for instance promoting anchorage-independent growth of NIH3T3 fibroblasts^2^. DENR, like MCTS1, is amplified in neuroendocrine prostate cancer according to publicly available databases (cBioPortal)^4^. Furthermore, de novo missense mutations in DENR were recently discovered in two unrelated patients with autism spectrum disorders, and DENR is important for proper migration and terminal branching of cortical neurons in the mouse^5^. Hence DENR and MCTS1 appear to play a role in cancer biology and in neurobiology.

DENR and MCTS1 form a complex that binds the 40S ribosomal subunit^1^. We recently showed that DENR and MCTS1 promote a process termed translation reinitiation^6^. During cap-dependent translation, ribosomes are recruited to the 5′ cap structure of mRNAs, and they then scan 5′ to 3′ until they meet an AUG initiation codon in a strong Kozak sequence context. On transcripts containing upstream Open Reading Frames (uORFs) with strong Kozak sequences (stuORFs), this presents a problem because ribosomes initiate translation on the stuORF, thereby consuming factors such as the initiator Met-tRNA^Met^_i_. Hence to translate the main downstream open reading frame, ribosomes need to terminate translation of the stuORF and reinitiate translation downstream^7, 8^. This process is not well understood, but likely requires recycling of ribosomal subunits, continued association with the mRNA, renewed scanning, and renewed recruitment of Met-tRNA^Met^_i_. We previously showed that DENR and MCTS1 promote translation reinitiation, and therefore are required specifically for translation of mRNAs containing stuORFs in their 5′UTRs, but they are dispensable for translation of mRNAs lacking stuORFs^6^. The Shatsky and Pestova groups have shown that DENR and MCTS1, or the structurally and functionally related protein Ligatin/eIF2D, have biochemical activities *in vitro* that are likely relevant for translation reinitiation - they can recycle post-termination ribosomal complexes and they can recruit Met-tRNA_i_^Met^ to the 40S ribosomal subunit in a non-canonical, eIF2-independent manner on viral mRNAs^9, 10^. Hence these activities might explain the mechanism by which DENR and MCTS1 promote translation reinitiation, although additional work will be necessary to unravel this mechanism in detail.

To understand the biological functions of DENR and MCTS1 in cancer and neurobiology, it is necessary to understand which transcripts in humans are dependent on DENR and MCTS1 for their efficient translation. We previously showed in Drosophila that stuORF containing transcripts are DENR•MCTS1 targets and that this class of genes is enriched for transcription factors, cell membrane receptors, and genes involved in neuron morphogenesis^6^. In this study we report the identification of very short stuORF-containing transcripts as targets for DENR•MCTS1 in human cells, and find that this class of transcripts is enriched for neuronal genes.

## Results

### DENR and MCTS1 are required for optimal expression of a synthetic stuORF reporter

**Figure 1:**
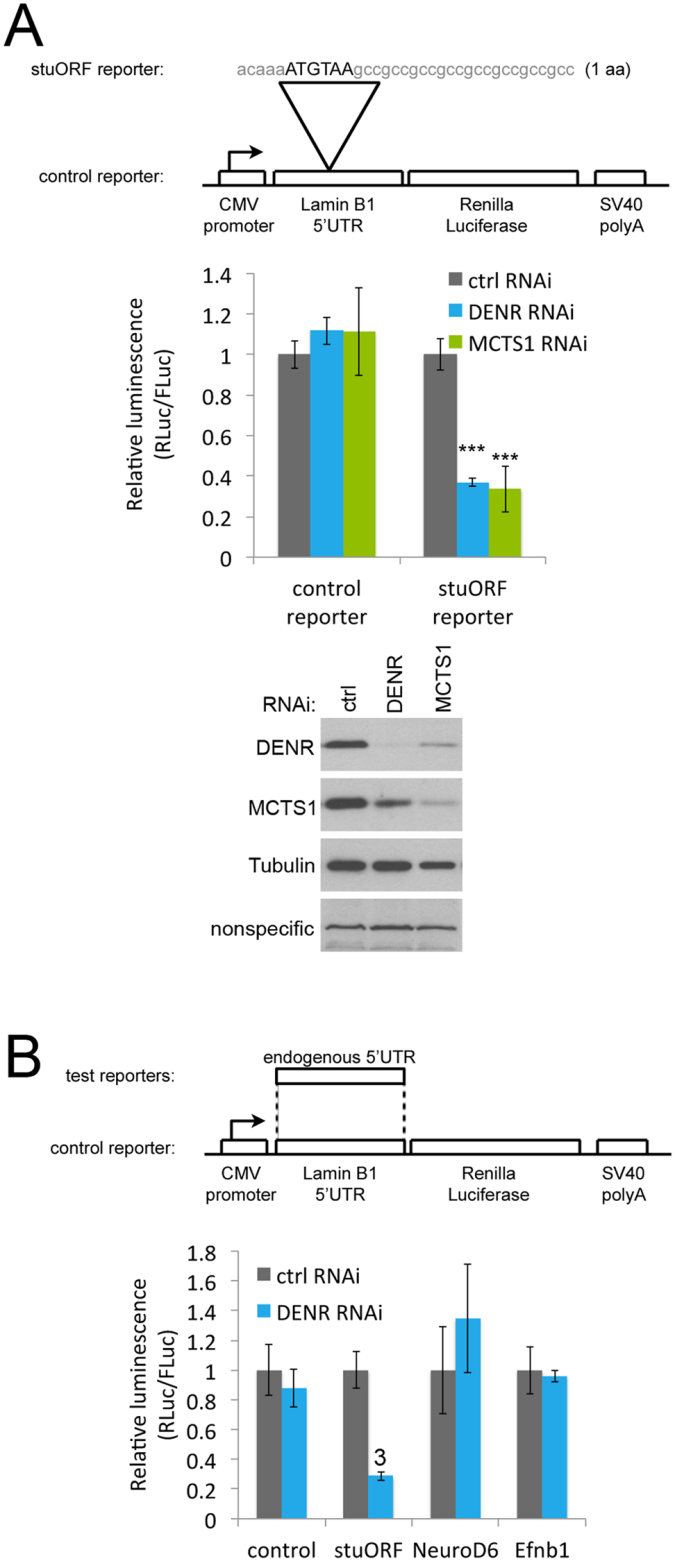
A synthetic reporter bearing a stuORF in the 5′UTR requires DENR and MCTS1 for optimal translation. (**A**) A luciferase reporter carrying a very short stuORF (strong uORF, a uORF with a strong Kozak sequence) in its 5′UTR specifically drops in translation compared to a control reporter, when either DENR or MCTS1 are knocked down (**A**). (Lower panel) Immunoblot controlling for DENR and MCTS1 knockdown efficiency. Immunoblot is cropped for presentation purposes. Uncropped version in Supplementary Fig. S5. (**B**) Mouse transcripts containing stuORFs were identified bioinformatically using the same parameters as those used previously in Drosophila^6^. The 5′UTRs of these transcripts, however, are not sensitive to DENR knockdown. The increase in NeuroD6 upon DENR knockdown is not statistically significant. Error Bars: Std. dev. ^3^Student ttest p ≤ 0.001 relative to ctrl RNAi.

We previously showed that expression of a reporter bearing a Drosophila 5′UTR with a synthetic strong-Kozak upstream Open Reading Frame (stuORF, with sequence acaaaATGTAA) is down-regulated in human cells upon DENR knockdown^5^. To test whether this effect is dependent on the context of the Drosophila 5′UTR, or whether it also works in the context of a human 5′UTR, we constructed a control Renilla Luciferase (RLuc) reporter bearing the human Lamin B1 5′UTR, which has no upstream Open Reading Frames (Fig. 1A). From this, a ‘stuORF reporter’ was generated by introducing the sequence acaaaATGTAA encoding for only 1 amino acid and having a strong Kozak sequence (Fig. 1A). As expected, knockdown of either DENR or MCTS1 (knockdown efficiency controls shown in lower panel of Fig. 1A) did not reduce expression of the control reporter, but reduced expression of the stuORF reporter (Fig. 1A). (Of note, we consistently see that knockdown of either DENR or MCTS1 leads to loss of the other protein as well, Fig. 1A. This is something we observe in both Drosophila^6^ and in human cells using multiple independent siRNAs targeting either DENR or MCTS1, excluding off-target effects. This co-dependence is often observed when several proteins form one functional complex, e.g. ref. 11). Reduced expression of the stuORF reporter was rescued by re-introducing siRNA-resistant versions of DENR or MCTS1 (Supplementary Fig. S1A,B), indicating the effect is specific. Unlike knockdown of DENR or MCTS1, knockdown of the structurally and functionally related protein Ligatin/eIF2D^7, 9, 10^ did not cause a drop in stuORF reporter activity (Supplementary Fig. S1C-C’), perhaps due to the fact that eIF2D is present in HeLa cells at much lower stoichiometry compared to DENR or MCTS1^12^. Hence we focus here on DENR and MCTS1.

To search for mammalian transcripts that depend on DENR•MCTS1 for their efficient expression, we searched the mouse transcriptome for stuORF-containing transcripts using the same parameters that previously successfully identified DENR-dependent transcripts in Drosophila^6^, taking into account the uORF Kozak sequence strength, but not the length of the uORF. This identified NeuroD6 and Efnb1, which have 14 and 3 stuORFs in their 5′UTRs respectively, as strong candidates. Surprisingly, however, reporter constructs bearing the NeuroD6 and Efnb1 5′UTRs were not sensitive to DENR knockdown (Fig. 1B). In sum, DENR and MCTS1 are required in both Drosophila and mammals for efficient expression of a synthetic stuORF-containing reporter, however the endogenous mammalian transcripts that are DENR-dependent likely have different features compared to the Drosophila ones.

### Effect of stuORF length and Kozak strength on DENR•MCTS1-dependence

**Figure 2:**
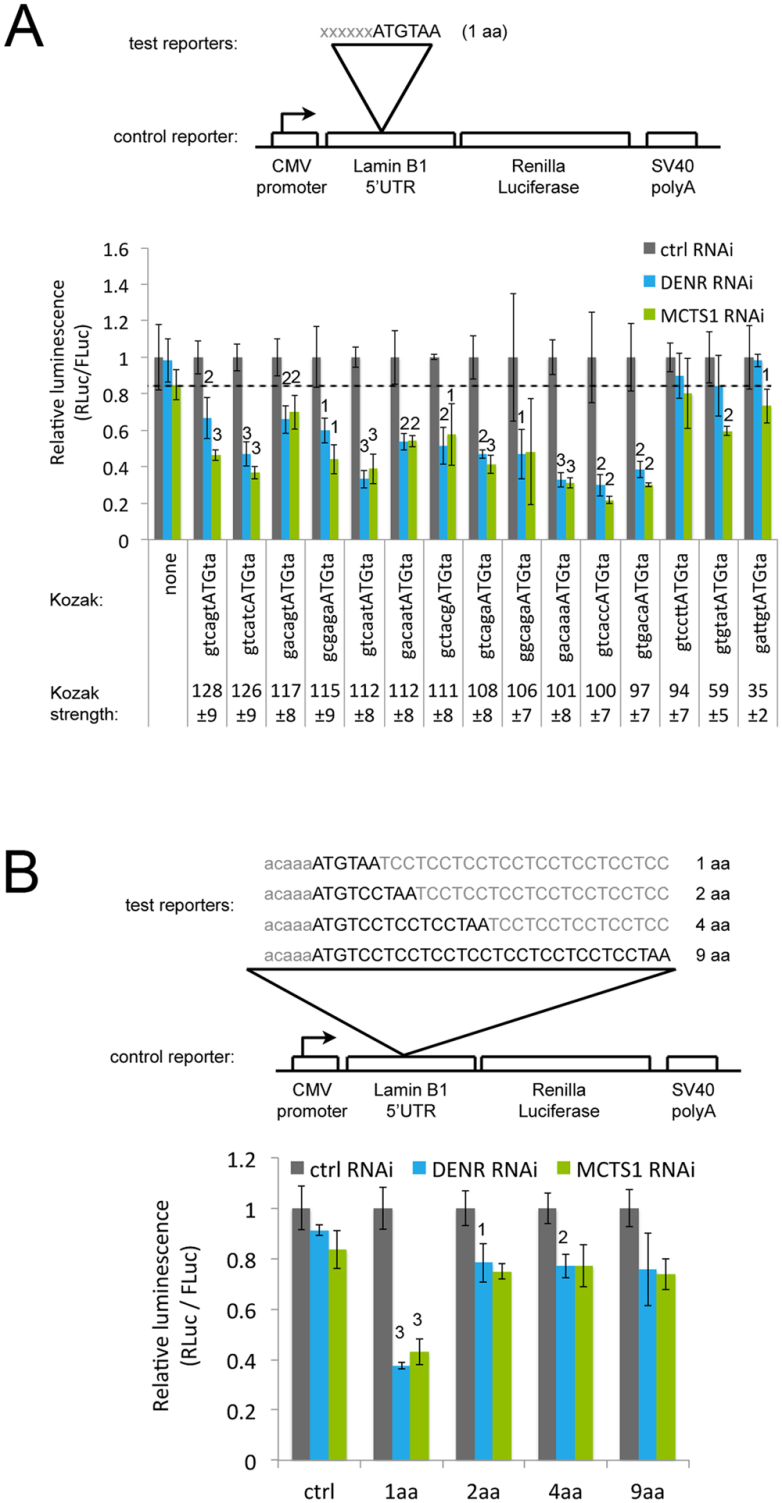
: Influence of the stuORF kozak sequence and stuORF length on DENR dependence. (**A**) Only transcripts bearing uORFs with strong Kozak sequences require DENR and MCTS1 for efficient translation. uORFs with Kozak sequences of various strengths were cloned into the 5′UTR of a control reporter, and tested for expression upon knockdown of DENR (blue) or MCTS1 (green). Kozak strengths are derived from^13^. uORFs with strengths below 95 do not impair translation upon DENR or MCTS1 knockdown. The dotted line serves as a visual aid to compare the constructs on the far right of the chart to the control reporter on the far left. Student ttest ^1^p ≤ 0.05, ^2^p ≤ 0.01, ^3^p ≤ 0.001 relative to ctrl RNAi. (**B**) The ability of DENR•MCTS1 to promote translation depends on stuORF length. (Top) Schematic diagram showing the stuORFs of various lengths that were cloned into the 5′UTR of a control reporter. (Bottom) Only very short stuORFs that are 1aa in length (ATGTAA) drop strongly upon DENR knockdown. Student ttest ^1^p ≤ 0.05, ^2^p ≤ 0.01, ^3^p ≤ 0.001 relative to ctrl RNAi and ctrl reporter. Error Bars: Std. dev.

We previously found that the degree of down-regulation of reporter expression upon DENR or MCTS1 knockdown in Drosophila depends on two parameters: the strength of the uORF Kozak sequence and the length of the uORF. We therefore tested the dependence on these two parameters in human cells. Noderer and colleagues previously used a sequencing-based method to determine the relative strength of all Kozak sequences in human cells^13^, yielding scores from 18 to 120 with half of all Kozak sequences scoring below 90. We selected a panel of sixteen different Kozak sequences and cloned them upstream of a 1 amino acid uORF (Fig. 2A). Expression of all reporters carrying uORFs with strong Kozak sequences (scoring above 97) was generally impaired upon DENR knockdown (blue bars, Fig. 2A, non-normalized values in Supplementary Fig. S2A). In contrast, uORFs with weaker Kozak sequences (scores below 94) did not cause expression of the reporter to be DENR dependent (Fig. 2A). This is in agreement with our previous findings in Drosophila that DENR and MCTS1 are specifically required to promote expression of transcripts bearing uORFs with strong Kozak sequences. Based on previous results^6^, this is likely because uORFs with weak Kozak sequences do not cause the ribosome to initiate translation, and therefore also do not require DENR•MCTS1 to promote translation re-initiation on the main downstream ORF. Similar results were obtained with MCTS1 knockdowns (green bars, Fig. 2A). We previously found^6^ that the presence of multiple copies of a stuORF in the 5′UTR of a reporter causes translation of the reporter to become more dependent on DENR and MCTS1. To test this in human cells, we generated reporters with 1, 2 or 3 copies of a moderately strong uORF and found that the effect size is small, but there is a tendency towards increased DENR or MCTS1 dependency with increasing stuORF copy number (Supplementary Fig. S2B-B’).

We next investigated the dependence on stuORF length. To this end, we cloned into the control reporter stuORFs (with a strong Kozak sequence) with upstream Open Reading Frames of different lengths (from 1 a.a. to 9 a.a. Fig. 2B). As observed in Drosophila, the translation of reporters carrying very short stuORFs is more dependent on DENR•MCTS1 than the translation of reporters with longer stuORFs (Fig. 2B). In contrast to Drosophila, however, where also 2 a.a. and 4 a.a.-long stuORFs are regulated by DENR•MCTS1, the dependence on stuORF length in human cells is very strong and essentially restricted to stuORFs of 1 a.a. in length. We also tested stuORFs containing the second strongest Kozak sequence identified by Noderer *et al*. genome-wide^13^ (Supplementary Fig. S2C-C’). Since this Kozak sequence contains a G at position +4, it cannot be tested with a 1 a.a. stuORF, which necessitates a T at +4 for the stop codon. Also in this context, the stuORF with a length of 2 amino acids imparted little to no DENR•MCTS1 dependence (Supplementary Fig. S2C’). Hence in both Drosophila and Human cells, the effect of DENR•MCTS1 on expression of a transcript depends on the strength of the uORF Kozak. The dependence on stuORF length in Drosophila and in humans, however, is qualitatively similar but quantitatively different, with essentially only 1 a.a. long stuORFs imparting DENR•MCTS1 dependence in the human.

### Identification of DENR•MCTS1-dependent human transcripts

**Figure 3:**
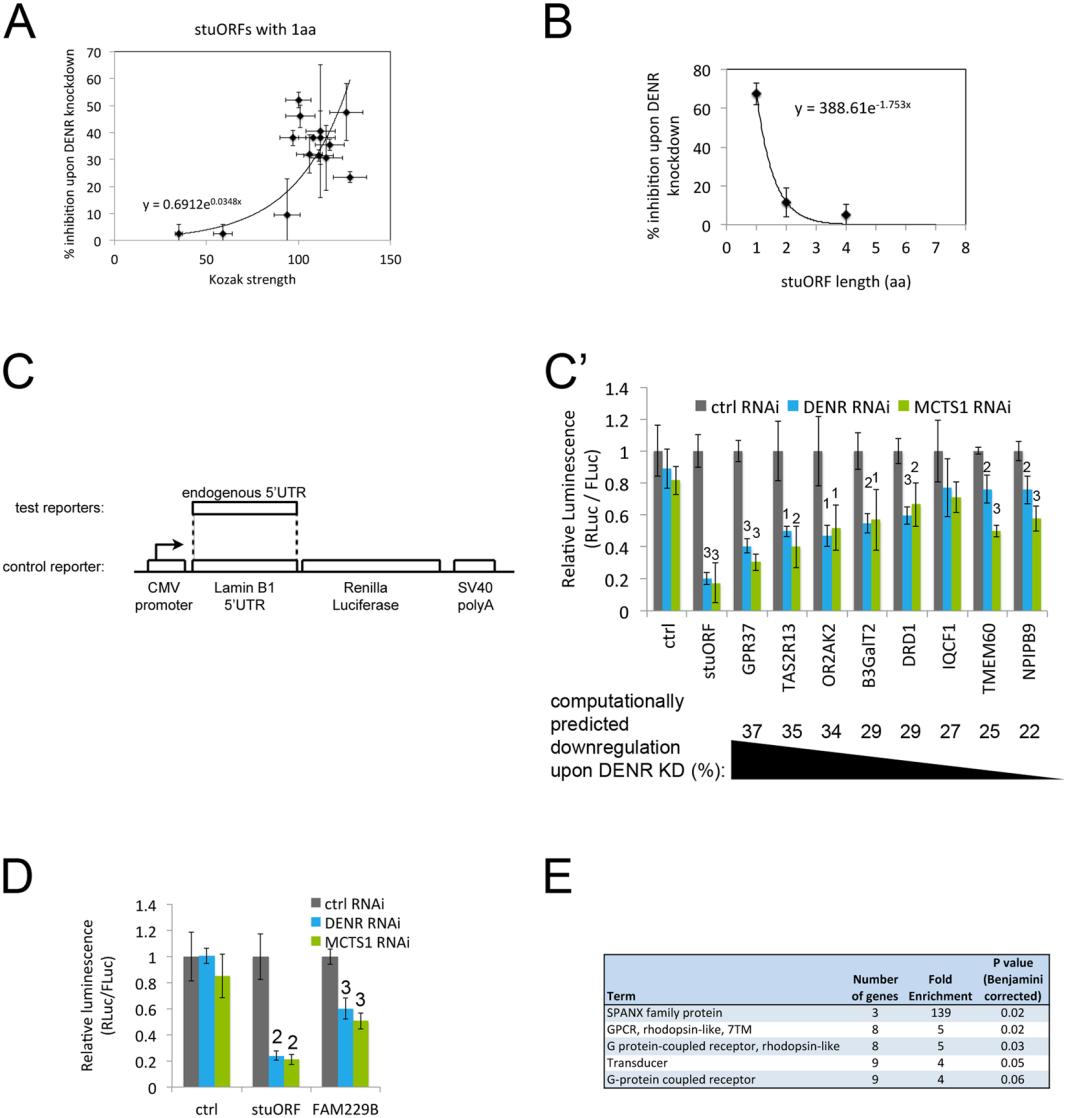
Identification of DENR and MCTS1-dependent human transcripts. (**A**,**B**) Compiled data summarizing the drop in translation of luciferase reporters upon DENR•MCTS1 knockdown, and the dependence of this translation drop on stuORF Kozak strength (**A**) or length (**B**). The indicated curves show the exponential regressions used to calculate DENR•MCTS1 dependence transcriptome-wide. (**C**-**C’**) Identification of human genes whose translation is DENR•MCTS1 dependent. 5′UTRs of human genes identified computationally as having very short stuORFs were cloned into luciferase reporter vectors (**C**) and tested for expression upon knockdown of DENR or MCTS1 (**C’**). Genes are arranged in order of decreasing predicted DENR•MCTS1 dependence. Student ttest ^1^p ≤ 0.05, ^2^p ≤ 0.01, ^3^p ≤ 0.001 relative to ctrl RNAi. (**D**) ‘Borderline’ target genes such as FAM229B, with a predicted down-regulation of 22% upon DENR knockdown, are also regulated if DENR or MCTS1 knockdown is efficient. Student ttest ^1^p ≤ 0.05, ^2^p ≤ 0.01, ^3^p ≤ 0.001 relative to ctrl RNAi. (**E**) Enrichment analysis for Gene Ontology using DAVID^14^ of human very short stuORF containing genes identifies GPCRs as an enriched class. Error Bars: Std. dev.

Using the results described above with synthetic uORF reporters, we searched the human transcriptome for transcripts predicted to be dependent on DENR•MCTS1 for their translation. The data from all our luciferase assays are summarized in Fig. 3A and B where Fig. 3A shows the dependence on Kozak strength and Fig. 3B on stuORF length. We fit the data to exponential curves and used these curves to predict the DENR•MCTS1-dependence for all human transcripts (Supplementary Table S1, and summarized per gene in Supplementary Table S2). We also tried a “Kozak-strength dependence curve” (Fig. 3A) that rises exponentially up to a Kozak strength of 90, and then flattens out and remains equal for all Kozak sequences with scores above 90, however this did not make a difference compared to the exponential dependence presented here. We then sorted all transcripts based on their predicted DENR•MCTS1 dependence, and selected several transcripts with a range of predicted down-regulation upon DENR knockdown from 37%, the maximum genome-wide, to 22%, for testing (Supplementary Table S3). We cloned the endogenous 5′UTRs of these genes into a reporter vector (Fig. 3C) and found that expression of most of these reporters is indeed DENR and MCTS1 dependent (Fig. 3C’). Furthermore, the degree of drop in expression upon DENR or MCTS1 knockdown roughly correlates with the prediction, thereby validating the predictions. The drop in luciferase activity upon DENR knockdown was not accompanied by a drop in reporter mRNA levels (Supplementary Fig. S3A-A’), consistent with a translational impairment. (The DRD1 5′UTR reporter contains an endogenous intron, which is spliced out *in vivo* when the reporter is transfected into HeLa cells, Supplementary Fig. S3B-B’). For some 5′UTRs such as those of TMEM60 and NPIPB9, knockdown of MCTS1 yields a stronger effect than knockdown of DENR (Fig. 3C’). One possible explanation is that the MCTS1 knockdown is more efficient (e.g. see also Fig. 2A). Reporters for genes predicted to be mildly DENR•MCTS1-dependent (e.g. FAM229B, predicted to be 22% down-regulated upon DENR knockdown), are also responsive to efficient DENR or MCTS1 knockdown (Fig. 3D). In sum, using a conservative threshold of predicted theoretical downregulation of 15% upon DENR•MCTS1 knockdown, this identifies a list of 104 genes as putative DENR targets (Supplementary Table S4), eleven of which have Drosophila orthologs that are also DENR targets (Supplementary Table S5). Of note, this table was generated by requiring that all transcript isoforms of a gene (which may differ in their 5′UTRs) are at least 15% downregulated upon DENR•MCTS1 knockdown. Additional genes exist for which individual transcript isoforms are predicted to be DENR•MCTS1 dependent, as listed in Supplementary Table S1. Both visual inspection of this target list, as well as a Gene Ontology enrichment analysis using DAVID^14^ revealed a very strong enrichment for G-protein coupled receptors in the target list (Fig. 3E). Indeed, many of the predicted DENR•MCTS1 are neuron-specific GPCRs, such as the taste receptor TAS2R13 or the olfactory receptor OR2AK2 (Fig. 3C’). Hence very short stuORF-containing targets of DENR and MCTS1 may be important in the context of neurobiology.

### GPR37 protein levels drop upon DENR•MCTS1 knockdown

**Figure 4:**
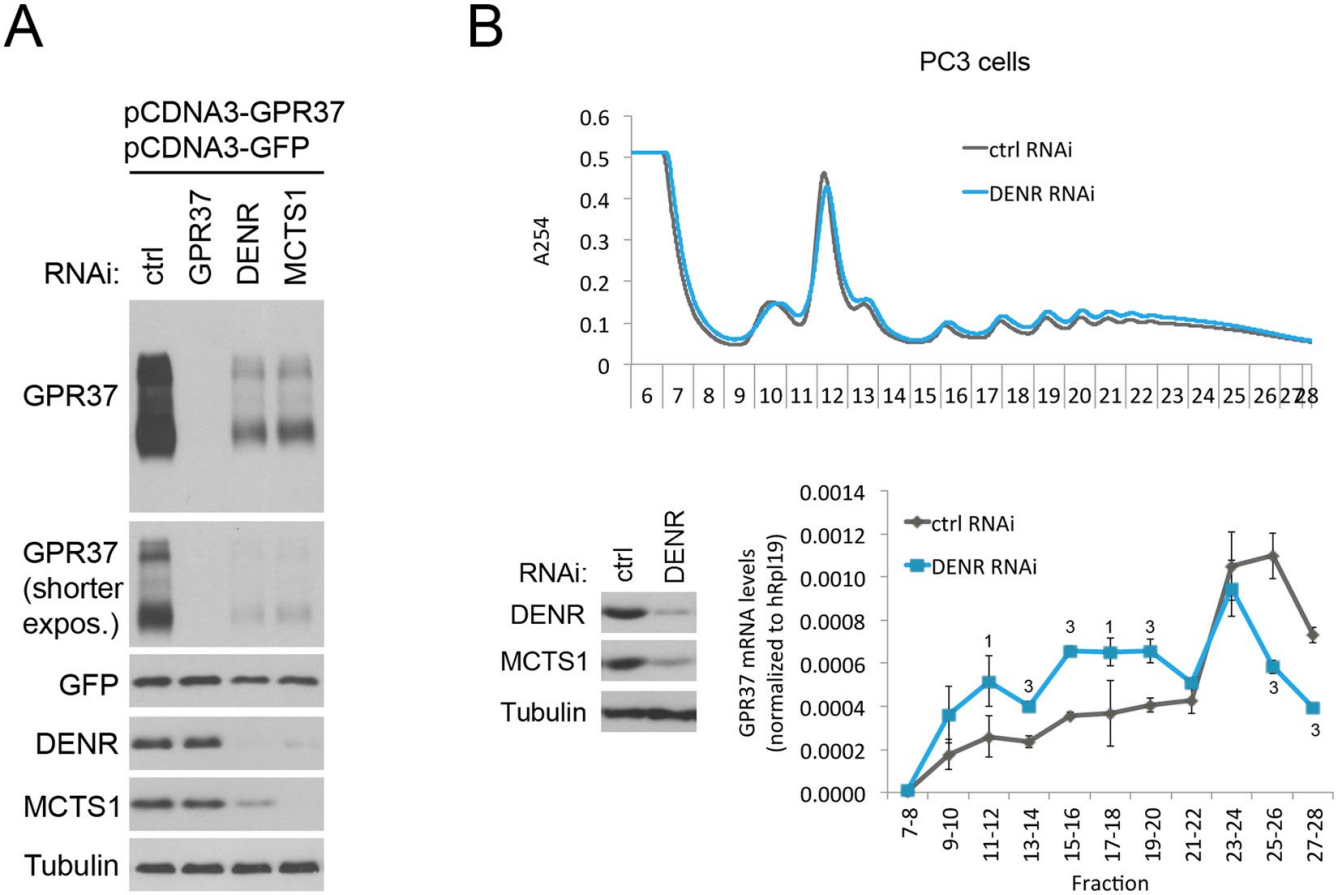
GPR37 protein expression is reduced upon knockdown of DENR or MCTS1. (**A**) GPR37 protein expression is reduced upon knockdown of DENR or MCTS1. The GPR37 transcript including 5′UTR, open reading frame, and 3′UTR, was cloned downstream of a constitutive CMV promoter, and co-transfected into HeLa cells together with a GFP expression construct bearing the same CMV promoter as a normalization control. GPR37 and GFP proteins were detected by immunoblots following the indicated siRNA-mediated knockdowns. Immunoblot is cropped for presentation purposes. Uncropped version in Supplementary Fig. S6. (**B**) Endogenous GPR37 mRNA shifts into lighter polysome fractions in PC3 cells upon DENR knockdown. (Top) Polysome profiles of PC3 cells treated with control or DENR siRNA. (Bottom left) Control immunoblot confirms DENR knockdown was efficient. (Bottom right) Quantitative RT-PCR on indicated fractions from the polysome gradient for endogenous GPR37 mRNA, normalized to hRpL19 mRNA, reveals that upon DENR knockdown GPR37 mRNA shifts into lighter fractions that are less translated. Error Bars: Std. dev. Student ttest ^1^p ≤ 0.05, ^3^p ≤ 0.001 relative to ctrl RNAi.

We next sought to measure the effect of DENR or MCTS1 knockdown on endogenous protein levels for one of these targets. Unfortunately, however, despite extensively testing commercially available antibodies, we were not able to find an antibody and cell line combination that successfully detected endogenous protein (with specificity judged by siRNA-mediated knockdowns of the proteins of interest). We tested anti-GPR37, anti-ADRA2A, anti-C10ORF12, anti-HOXB4 and anti-LCA5 antibodies in multiple different cell lines (see Materials & Methods for a detailed list). This is likely because many of the DENR targets are expressed strongly in differentiated neurons or glia, and only at very low levels in most cell lines in culture. Hence, we resorted to two alternate approaches. Firstly, we cloned the entire GPR37 transcript (5′UTR, ORF, and 3′UTR) downstream of a constitutive CMV promoter (pCDNA3-GPR37), and expressed it in HeLa cells. We knocked down DENR or MCTS1 and detected GPR37 by immunostaining. As expected, knockdown of either DENR or MCTS1 led to a strong reduction in GPR37 protein (Fig. 4A). This approach has the advantage of normalizing for transcriptional effects, as judged by a similar GFP-expressing construct (pCDNA3-GFP) that was co-transfected as a normalization control and did not show a similar drop as GPR37 (Fig. 4A). The drop in GPR37 protein was not accompanied by a drop in GPR37 mRNA levels (Supplementary Fig. S4A) or in GPR37 protein stability, assayed by performing a cycloheximide chase experiment (Supplementary Fig. S4B-B’), consistent with DENR•MCTS1 having a translational effect of on GPR37. As a second approach, we looked at the distribution of endogenous GPR37 mRNA upon DENR knockdown in polysome gradients. In such gradients, mRNAs that are actively translated sediment into heavier fractions containing more ribosomes, compared to mRNAs of equal length that are less well translated. From publicly available expression databases^15^, we selected cell lines with comparatively high levels of GPR37 mRNA. By quantitative RT-PCR we identified prostate cancer PC-3 cells as having comparatively high levels of GPR37 transcript (Supplementary Fig. 4C). Although we could not detect GPR37 protein in PC-3 lysates by immunoblotting, quantitative RT-PCR was sensitive enough to detect GPR37 mRNA. We then treated PC-3 cells with either control or DENR siRNAs and generated polysome gradients from the cell extracts. Quantitative RT-PCR on the RNA obtained from the various fractions of these polysome gradients revealed that upon DENR knockdown endogenous GPR37 mRNA shifted into lighter fractions containing fewer ribosomes, in agreement with reduced translation (Fig. 4B). Finally, we tested whether knockdown of DENR or MCTS1 impairs the proliferation of HeLa cells, however the proliferation impairment is very mild (Supplementary Fig. S4D), consistent with the fact that the DENR target genes we report here are not expressed in HeLa cells.

## Discussion

We previously identified DENR and MCTS1 target transcripts in Drosophila by searching for transcripts that have many stuORFs of any length^6^. This approach was successful in Drosophila, likely because the Drosophila system is less stringent than the human one for stuORF length. This approach did not work, however, for mammalian transcripts, thereby necessitating the current study. We find that the Drosophila and human systems work in a qualitatively similar fashion: in both cases the effect of DENR or MCTS1 knockdown on translation depends on both the strength of the uORF Kozak sequence and the length of the uORF. The Drosophila and human systems, however, are quantitatively different in that only stuORFs with very short coding sequences, coding for 1 or maximally 2 amino acids, impart DENR•MCTS1 dependence in the human system. It is thought that translation reinitiation after a uORF requires translation initiation factors such as eIF4F, which remain transiently associated with the ribosome upon uORF start codon recognition and formation of the 80S ribosome. These factors, however, are thought to gradually dissociate from the ribosome while it is elongating^7^. Hence the capacity to reinitiate drops the longer the ribosome elongates. It is possible that these initiation factors dissociate more quickly from the ribosome in human cells compared to Drosophila cells, leading to efficient reinitiation in humans only after very short uORFs of 1 or 2 amino acids in length. This leads us to a list of only 104 predicted targets in humans, which is more restricted than what we found in flies. Interestingly, in both humans and in Drosophila, the list of putative target genes is enriched for cell membrane proteins (such as GPCRs) and for genes involved in neuronal biology, suggesting this is a conserved aspect of DENR•MCTS1 function.

The degree by which translation of a transcript drops upon DENR or MCTS1 knockdown depends on the strength of the uORF AUG context. uORFs with weak Kozak sequences (in Fig. 2A, Kozak strengths <95) do not impart DENR•MCTS1 dependence to a transcript, whereas uORFs with strong Kozak sequences (>95 in Fig. 2A) were generally impaired upon DENR knockdown. However, if we consider only the uORFs with strong Kozak sequences, and look at higher resolution, we do not see a clear correlation between Kozak strength and the degree of DENR dependence (Fig. 2A). For instance, our positive control ‘stuORF reporter’ with the very short stuORF sequence gacaaaATGtaa has a predicted Kozak strength of 101 ± 8, yet it causes a stronger drop in reporter translation upon DENR knockdown than other uORFs with stronger Kozak sequences (e.g. gacagtATGta with a strength of 117 ± 8). One possible explanation is that the measurement of Kozak strength by Noderer *et al*.^13^ does not have this level of precision, as suggested by the error intervals they provide. Alternatively, the sequence context of the uORF AUG also affects DENR dependence in additional ways that we do not yet understand.

DENR and MCTS1 are misexpressed or mutated in patients with cancer or autism spectrum disorders. By identifying the DENR•MCTS1 dependent transcripts in humans, this current study enables future work aimed at understanding how these proteins affect cancer and brain function. In the context of autism, it appears of relevance that many of the target genes are GPCRs that are selectively expressed in the central nervous system. For instance, one of the 5′UTRs that responds most strongly to DENR or MCTS1 knockdown is that of GPR37 (Fig. 3C’). GPR37 affects oligodendrocyte differentiation and myelination^16^, as well as dopamine uptake by neurons^17^ and mutations in GPR37 have been identified in autism patients^18^. Hence GPR37 may be an interesting target gene to follow-up in the future. Autism patients also have defects in sensory perception^19, 20^. Interestingly, amongst the most DENR•MCTS1 dependent genes are both taste and olfactory receptors TAS2R13 and OR2AK2 (Fig. 3). Since MCTS1 is overexpressed in several types of cancer, and MCTS1 overexpression drives the cell cycle and promotes anchorage-independent colony formation^1, 21, 22^, we were surprised that we did not find obvious oncogenes in the list of DENR•MCTS1 targets. Several explanations are possible. Firstly, cancer relevant genes may indeed be present in this list, which are not obvious at first sight. Secondly, the list presented here only includes genes for which all transcript isoforms are predicted to be DENR•MCTS1 targets. One thousand nine hundred fifty genes have at least one splice isoform that is predicted to be DENR•MCTS1 dependent for translation (Supplementary Table S1), and amongst these 1950 genes there are many that are cancer relevant. This is particularly relevant if the transcript isoform that is DENR•MCTS1 dependent is the only one expressed in a cell, or the only one with cancer-relevant function. Hence additional work will be required to decipher this. Finally, it is possible that DENR and MCTS1 also regulate additional classes of mRNAs via alternative mechanisms.

In sum, we identify here transcripts containing very short stuORFs as transcripts that require DENR and MCTS1 for optimal expression in human cells. This work will likely be a useful starting point for future studies analyzing the functional involvement of DENR and MCTS1 in cancer and autism spectrum disorders.

## Methods

### Plasmids

Renilla luciferase (RLuc) test reporters for luciferase assays were generated as follows: An internal SpeI restriction site in pRL-CMV Vector (Promega) was removed by cutting, blunting, and re-ligating to facilitate subsequent oligo clonings, yielding pSS350. The 5′UTR of human LaminB1 was amplified by PCR from HeLa cell cDNA using primers ccggaagcttGCCGCTCCGTGCAGCCTGAGAG and ccggTTCGAAgtCATggtggCGGGCGGCGGAGACAGCG and cloned into the pRL-CMV Spe-vector. To enable oligo clonings, SpeI and AgeI restriction sites were inserted into laminB1 5′UTR by oligos actagtGTGaccggtCCCTTTGTGCTGTAATCGAG and accggtCACactagtCAAAGGCGCGCGGGGGGGAA, yielding pSS372. This vector was used as the control reporter, and also served as a vector backbone for all subsequent oligo clonings with various Kozaks and uORFs.

The Firefly Luciferase (FLuc) normalization reporter was generated by removing the RLuc ORF from pRL-CMV by digestion with NheI and XbaI and replacing it with the FLuc ORF. In order to get a comparable normalization vector, the LaminB1 5′UTR was cloned upstream of the FLuc ORF via HindIII and NheI. This reporter, pSS411, served as normalization control in all luciferase assays.

For the very short stuORF test reporter the sequence ctagtGTGtccggacaaaATGTAAGCCGCCGCCGCCGCCGCCGCCGCCa^1^ was oligo cloned into the SpeI and AgeI sites of pSS372, yielding pSS373. This vector was used as a stuORF positive control. In this and all other cases, the uORFs have a stop codon that terminates the uORF upstream of the main ORF, hence the uORF does not form an in-frame fusion to the main ORF.

To test endogenous human 5′UTRs in luciferase assays, 5′UTRs were amplified from genomic DNA using oligos in Table 1 below, and cloned into the HindIII and BstBI sites of pSS350. For DRD1, this PCR product includes an intron. For FAM229B, GPR37, TMEM60 and B3GALT2, the individual 5′UTR exons were PCRed separately and combined by Gibson assembly into the reporter plasmid, yielding a ‘spliced’ 5′UTR lacking introns. For FAM229B, the last 14nt of the 5′UTR are not included in the reporter plasmid because they are on a separate exon, and do not contain any uORFs. The short 5′UTRs of IQCF1 and OR2AK2 were introduced by oligo cloning into the same vector using the oligos below.

**Table 1:** Sequences of oligos used for clonings.

Gene	Oligo sequence
GPR37	forward: CCGGAAGCTTGGGGTTGGAATCCCGC
	reverse: CCGGTTCGAAGTCATGGTGGGGCTTGGTGAGGGC
TAS2R13	forward: CCGGAAGCTTTCTGCCCATGGTGAAGAC
	reverse: CCGGTTCGAAGTCATGGTGGGTCAGAACAGAGAAAGTTCA
B3GALT2	forward: CCGGAAGCTTCGTTAAATTCTGCTCTGTCA
	reverse: CCGGTTCGAAGTCATGGTGGGTTGTAAATATCCAGTAGTGG
DRD1	forward: CCGGAAGCTTGTGCCCCGCGGGAACC
	reverse: CCGGTTCGAAGTCATGGTGGCTTCCTAAGAGAAAGCACA
TRIM42	forward: CCGGAAGCTTAATGCCAGCCTATGAGCTT
	reverse: CCGGTTCGAAGTCATGGTGGGGTGCCTACATGATTACCAC
TMEM60	forward: CCGGAAGCTTGTTTAAAGGTCGGTTGGC
	reverse: CCGGTTCGAAGTCATGGTGGTTAAACAGTTTAAAGA
FAM229B	forward: CCGGAAGCTTGCCGCGCAAGTGCACTT
	reverse: CCGGTTCGAAGTCATGGTGGCAAAAGAGGCTGAGTCT
IQCF1	forward: ccggaagcttATGGCTGGATGAACACAGATGTGAGGGGATTTGAACCATCTACAATCAATCCATATGTGACCA
	reverse: ccggttcgaagtcatggtggTGGTCACATATGGATTGATTGTAGATGGTTCAAATCCCCTCACATCTGTGTTCATCCAGCCAT
OR2AK2	forward: ccggaagcttTGCTCTAAACATGTAGATAGTTGCCAAACATGTAAGTGAATATTTATTTCTGAATGCCATGTCATTTTACTTTCTCTTAAGGGAAGTCAACATTATTAC
	reverse: ccggttcgaagtcatggtggGTAATAATGTTGACTTCCCTTAAGAGAAAGTAAAATGACATGGCATTCAGAAATAAATATTCACTTACATGTTTGGCAACTATCTACATGTTTAGAGCA
NPIPB9	forward: ccggaagcttTGTGCCAAATCATTCA
	reverse: ccggttcgaagtcatggtggGTCACTGACTTTACATGCTGCTGCAG

The Kozak sequence CCACC immediately upstream of Firefly and Renilla ORFs was maintained in all laminB1 reporters as well as in the human 5′UTR constructs.

The expression vector pCDNA-GPR37 was obtained by amplifying the entire genomic transcribed region of GPR37 from human genomic DNA via multiple overlapping PCRs and subsequently cloning it into pCDNA3 bearing a CMV promoter via Gibson assembly (New England Biolabs). The ‘top’ oligo for the first PCR fragment and the ‘bottom’ oligo for the last PCR fragment were: ACTAGTAACGGCCGCCAGTGTGCTGGAATTCGGGGTTGGAATCCCGC and GGGCCCTCTAGATGCATGCTCGAGCGGCCGCTTGTATCTTTAAGGCAAT. For the ‘normalization control’ CMV-GFP plasmid, the GFP ORF was amplified and cloned into pCDNA3.

### Transfections and luciferase assays

Gene knockdowns were performed using RNAiMax (Thermo Fischer Scientific) according to the manufacturer’s reverse transfection protocol in Opti-MEM (Thermo Fischer Scientific) with siGENOME siRNA pools (Dharmacon) for DENR, MCTS1 and GPR37 (Dharmacon), or Silencer Select Negative Control siRNA (Ambion). For reconstitution experiments MCTS1 siRNA3 and a pool of DENR siRNAs 1, 2 and 4 were used. siRNA sequences are provided in Table 2.

**Table 2:** Sequences of siRNAs used.

Gene	siRNA sequences
DENR	siRNA1: GCAGAUUUAUCCAUCGAGA
	siRNA2: GGUAAUGUCAAGUGGAGUA
	siRNA3: GACAAUGGUUAGAGAAGAA
	siRNA4: CAAGUUAGAUGCCGAUUAC
MCTS1	siRNA1: CCGAUGCCAUGAACAUAUA
	siRNA2: UAUCAUGUGUCCAGGCUUA
	siRNA3: CAAAGGAAUUGGCAUUGAA
	siRNA4: UAAGAAAGAUCCUGUCAAA
GPR37	siRNA1: GCAGAAAGGUGCAUUAUUA
	siRNA2:UAAGAUCUCUCCUGAUUUA
	siRNA3: ACAAUGGACCUCCUUAAUA
	siRNA4: GGUGGGAGCUCUAUUGUUA
eIF2D	siRNA1: GCACAAAGAGCGUCUAAUA
	siRNA2: GAACGAUCGUCAUUAACUA
	siRNA3: ACAAAUGGAUGAGCUGUUA
	siRNA4: GAACUGAUCAAGUCUCUGA

After distribution in a 96well format for luciferase assays, or 24 well formats for immunoblots, Hela cells were seeded in normal DMEM growth medium (Thermo) and 10% FCS (Sigma) onto the mix. Gene knockdowns were allowed to take place for four days at 37 °C. For luciferase assays the cells were transfected in quadruplicate with Effectene (Qiagen) on day three with the corresponding renilla and firefly reporters. For reconstitution experiments (Figure S1A,B), siRNA-insensitive constructs of MCTS1 or DENR or with control vector were also transfected in addition to the luciferase reporters. In the MCTS1 reconstitution experiment DENR had to be co-expressed in all conditions in order to achieve full recovery of complex activity. Insensitivity to siRNA was obtained by the following silent mutations: for MCTS1 - c.480A- > G, c.483A- > C, c.486T- > C, c.489C- > G, c.492T- > C, c.495A- > G; for DENR - c.63T- > C, c.66A- > G, c.67C- > T, c.69G- > A, c.72C- > T, c.75T- > C, c.78C- > T, c.81T- > C. In the reconstitution setting triplicates were performed. Knockdown and reconstitution of protein levels were verified by Western Blot in parallel. Five hours before performing the luciferase assay, the medium was replaced with fresh medium. For the dual-luciferase assay, medium was removed and cells lysed with Passive Lysis Buffer (Promega). After 20 min incubation the suspension was analyzed using the Dual Luciferase reporter assay (Promega).

### Quantitative RT-PCR

Sequences of oligos used for quantitative PCR are provided in Table 3.

**Table 3:** Sequences of oligos used for Q-RT-PCR.

Gene	oligo sequences
GPR37	oligo 1: ATCTTCCACGAGCTGACCAAG
	oligo 2: AAAGTGGTGACTCCCAGAGAAG
Renilla luciferase	oligo 1: CGGACCCAGGATTCTTTT
	oligo 2: TTGCGAAAAATGAAGACCT
hRpL19	oligo 1: ATGTATCACAGCCTGTACCTG
	oligo 2: TTCTTGGTCTCTTCCTCCTTG
DRD1 splicing	oligo 1: AAGGCAAGGCGTTTGGA
	oligo 2: TCTGGTGGTGACAGGAGAT

### GPR37 expression and immunoblots, and antibodies

After three days of knockdown as described above, HeLa cells in 24 or 12-well format were transfected with expression vectors pCDNA-GFP and pCDNA- GPR37 or pCDNA- GPR37 alone with Effectene (Qiagen) and grown for 20 hrs at 37C. For harvesting, cells were scraped in the medium and briefly centrifuged. The cell pellet was solubilized in Laemmli sample buffer together with cOmplete Protease Inhibitors (Roche) and Benzonase (Merck) and incubated for 30 min at room temperature. The solution was then briefly boiled and protein expression was assessed by PAGE and Immunoblotting. For assessing GPR37 protein stability cells were kept untreated or treated with 50 μg/mL cycloheximide for 2 hours prior to lysis. Antibodies were purchased from Sigma (DENR: mouse monoclonal WH0008562M1, tubulin: mouse monoclonal T9026, Ligatin/eIF2D: rabbit polyclonal HPA028220) and Abcam (GPR37: rabbit polyclonal ab166614) or obtained by immunizing guinea pigs with purified MCTS1 or GFP protein.

### Antibodies that did not work on endogenous proteins, as advertised by the producing companies

Guided by immunoblot images provided on the websites of companies selling antibodies, we tested the antibodies listed in Table 4 on immunoblots of the following cell lysates, but either did not obtain a band of roughly the correct size, or the band did not decrease in intensity upon siRNA-mediated knockdown of the corresponding genes.

**Table 4:** Antibodies tested which did not give specific bands in our hands.

Antigen	Antibody	Cell line lysates
GPR37	PA5-13413 (Life Technologies)	HeLa and T98G
GPR37	sc 27548 (Santa Cruz)	HeLa, T98G and MKN45
GPR37	sc 390110 (Santa Cruz)	HeLa and T98G
GPR37	ab166614 (Abcam)	T98G and MKN45 (this antibody worked on overexpressed GPR37 protein, and is used in Fig. 4)
ADRA2A	NBP1-9562 (Novus Biologicals)	HeLa
ADRA2A	NBP1-67832 (Novus Biologicals)	HeLa
C10ORF12	NBP1-70426 (Novus Biologicals)	Caco-2 and HEK293
HOXB4	00003214-A01 (Abnova)	HEK293
LCA5	NBP1 55416 (Novus Biologicals)	Caco-2 and HepG2

### Calculation of DENR•MCTS1 dependence

The predicted reduction in expression of a transcript upon DENR or MCTS1 knockdown was calculated as follows. For each upstream Open Reading Frame (uORF) in the transcript, the strength of the uORF Kozak was derived from ref. 13 and the uORF length (in terms of number of amino acids coded) was counted. Based on the data shown in Fig. 3A and B, the contribution of the individual uORF towards downregulation of the transcript was calculated as:

$$ \% \,downregulation=(0.6912\cdot {e}^{0.0348\cdot kozak\_strength})\cdot (\frac{388.61}{67.3}\cdot {e}^{-1.753\cdot uORF\_length})$$

Based on results from ref. 1, the contributions of the individual uORFs of a transcript were added together to arrive at the combined score for the entire transcript. These calculations were performed on all transcripts present in ENSEMBL for the human genome (release GRCh38.p5).

The algorithm which does not successfully predict DENR•MCTS1 targets (which was used to obtain NeuroD6 and Efnb1 as putative targets in Fig. 1B) ignored uORF length, and scored uORFs according to the consensus Kozak sequence, so that a G at position +1 yielded a score of 1, and if that was not the case, an A or C at position −3 yielded a score of 0.4 and a G or T at position −3 yielded a score of 0.1. Scores for individual uORFs in one 5′UTR were then added to arrive at a combined score for the entire transcript.

### Polysome profiling of PC-3 cells

PC-3 cells were transfected with control or DENR siRNA using RNAiMax as described above. After three days, cells where treated with cycloheximide (100 µg/ml) for 5 min at 37 °C, and then scraped off the dish and counted. The cells where then lysed in polysome buffer (15 mM Tris pH 7,5, 15 mM MgCl_2_, 300 mM NaCl, 1% Triton X-100, 2 mM ß-Mercaptoethanol, supplemented with EDTA free protease inhibitors and RNAse Inhibitors) and lysate from an equal number of cells was loaded on top of a 17,5–50% sucrose gradient. After ultracentrifugation at 4 °C at 35000 rpm for 2.5 hrs, gradient fractions where collected in a Biocomp gradient fractionator, and prepared for RNA extraction by adding an equal volume of Gough Solution II (10 mMTris-pH 7.5, 350 mM NaCl, 10 mM EDTA, 1% SDS,7 M Urea). After heating at 65 °C for 10 min, an equal volume of PCI (Phenol, Chloroform, Isoamyl﻿alcohol, 25:24:1) was added to the sample. After spinning, the aqueous phase was moved to a fresh tube and precipitated with 1,2x volumes of Isopropanol and 1 µg of Glycogen and precipitated o/n at −20 °C. After washing with 70% EtOH the sample was dried and reconstituted in water.

## Electronic supplementary material

Supplementary Information

Table S1

Table S2

Table S3

Table S4

Table S5
