# Supplementary material for: Identification of transcripts with short stuORFs as targets for DENR•MCTS1-dependent translation in human cells
Source: Sci Rep. 2017 Jun 16;7:3722. doi: 10.1038/s41598-017-03949-6 (PMC5473865; doi:10.1038/s41598-017-03949-6)
Supplement: Supplementary file 1 — Supplementary Information [file 41598_2017_3949_MOESM1_ESM.pdf]

Supplementary Information

Identification of transcripts with short stuORFs as targets for DENR•MCTS1-dependent translation in human cells

Sibylle Schleich, Julieta M. Acevedo, Katharina Clemm von Hohenberg and Aurelio A. Teleman

Contents

Supplementary Figure S1.. p. 2

Supplementary Figure S2.. p. 3

Supplementary Figure S3.. p. 4

Supplementary Figure S4.. p. 5

Supplementary Figure S5.. p. 6

Supplementary Figure S6.. p. 7

Supplementary Figure Legends .... p. 8

Schleich et al - Suppl. Figure S1

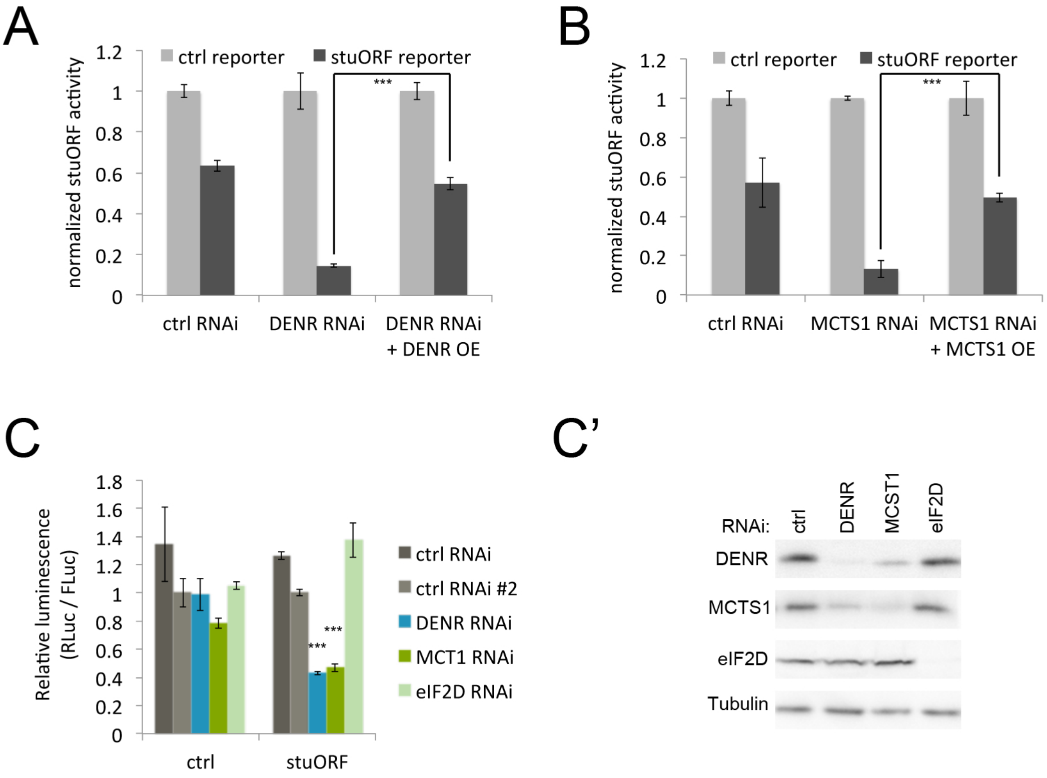

Schleich et al - Suppl. Figure S2

A

Non-normalized values for Figure 2A.

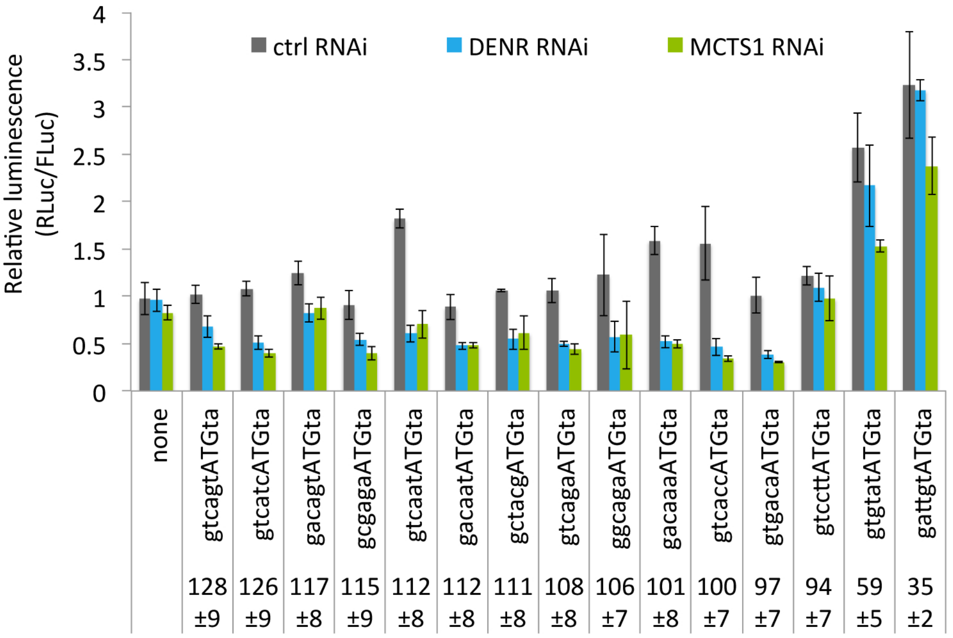

B

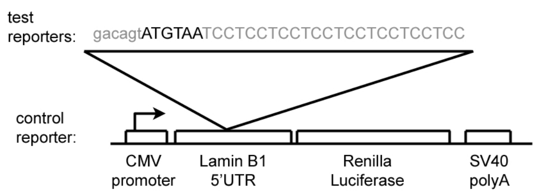

C

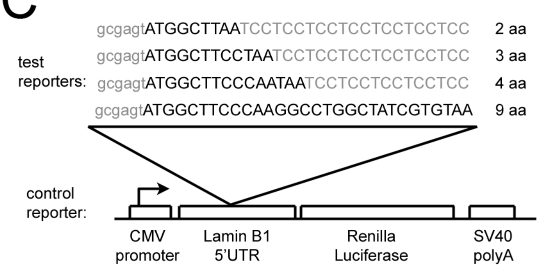

B'

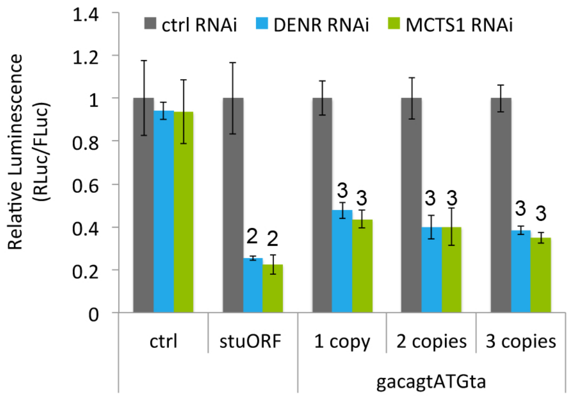

C'

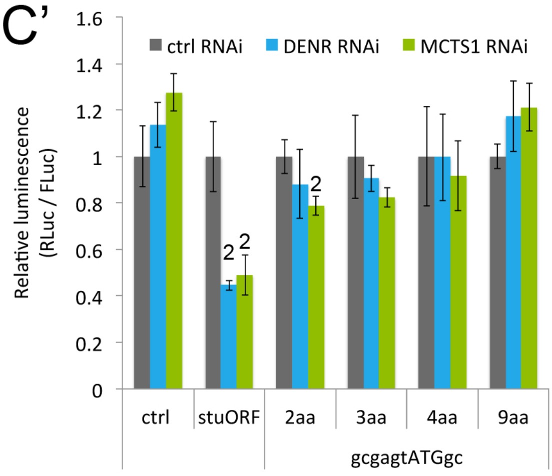

Schleich et al - Suppl. Figure S3

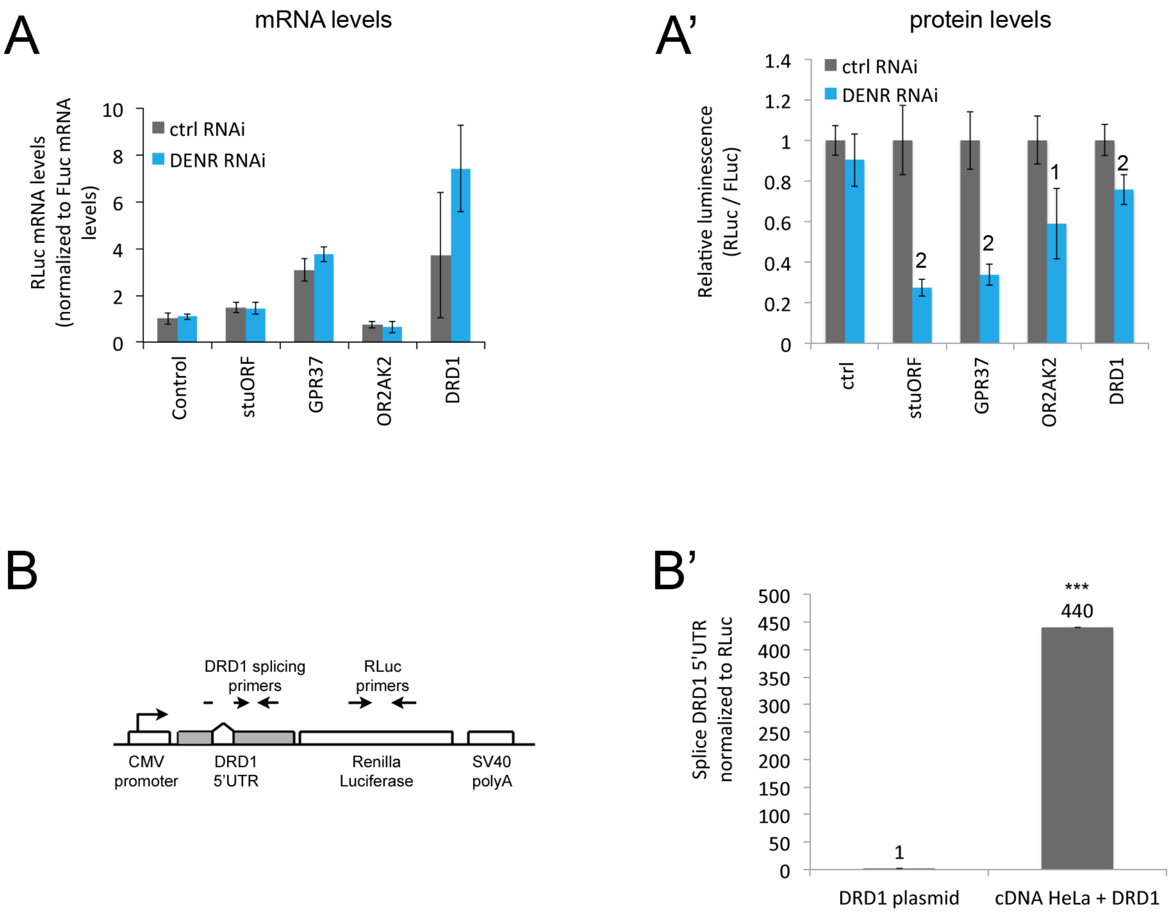

Schleich et al - Suppl. Figure S4

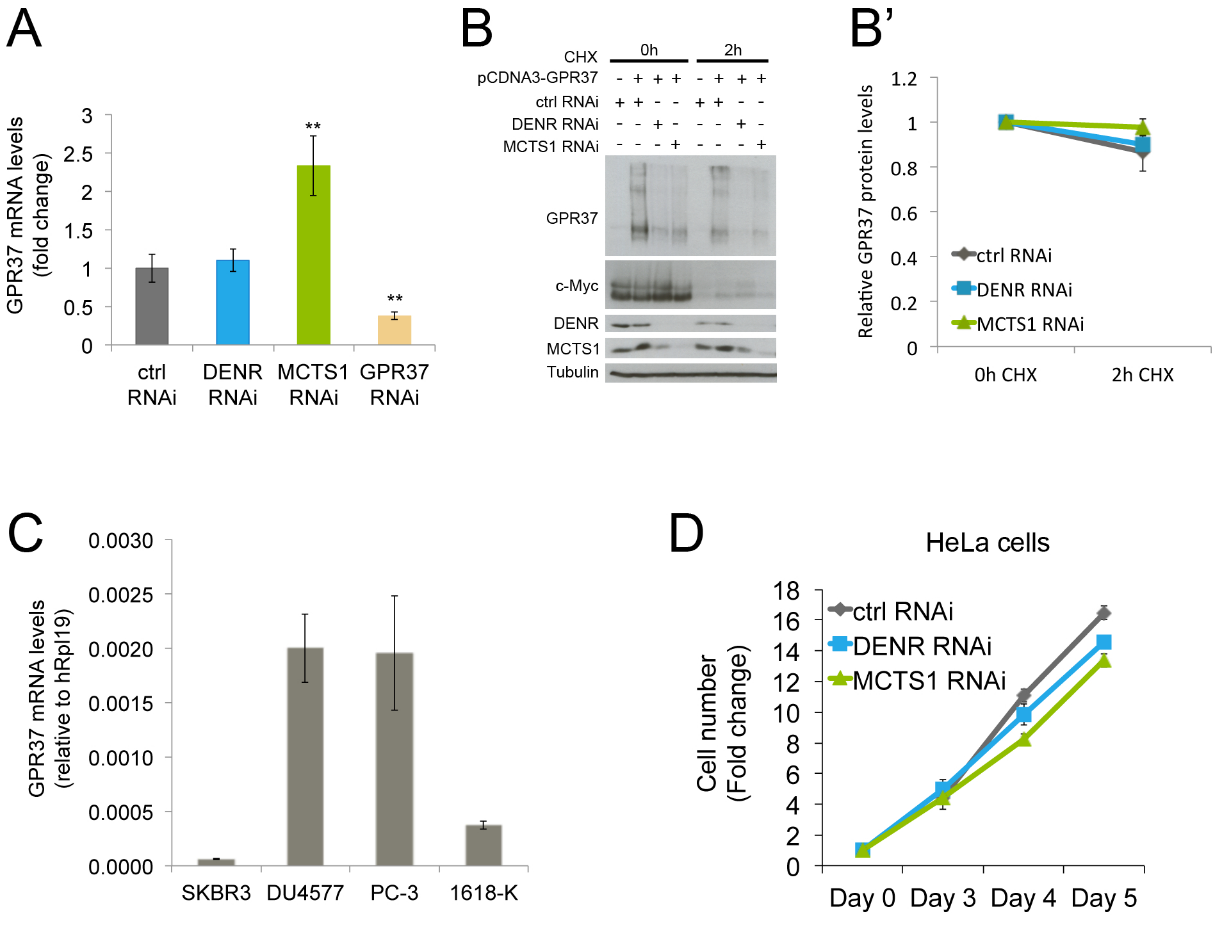

Schleich et al - Suppl. Figure S5
Uncropped blots for Main Figure 1A

DENR

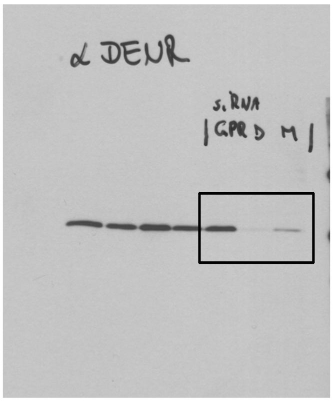

Tubulin

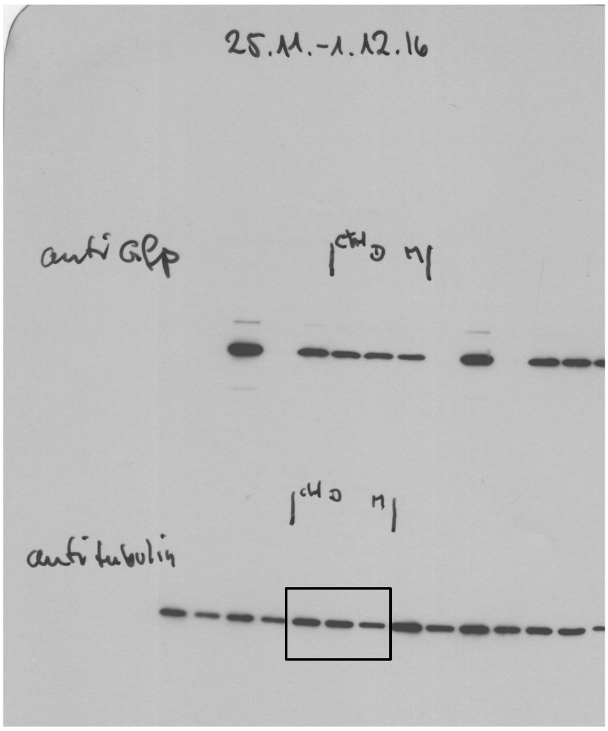

MCTS1 &
non-specific

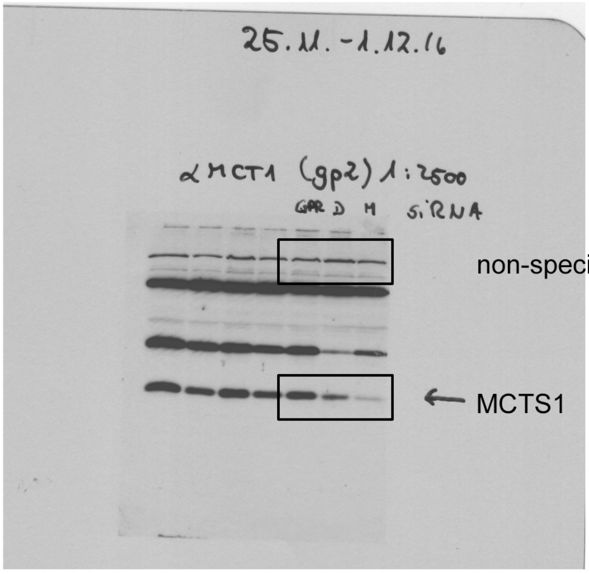

non-specific

← MCTS1

Schleich et al - Suppl. Figure S6
Uncropped blots for Main Figure 4

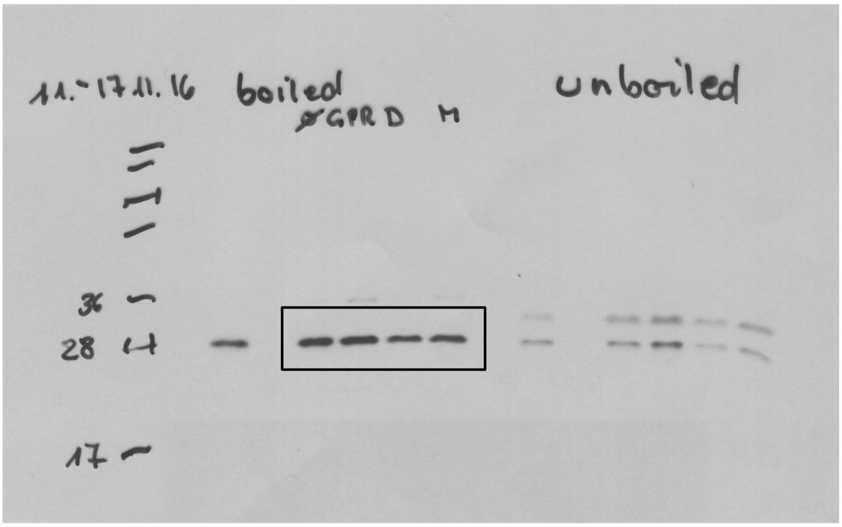

GFP

Tubulin

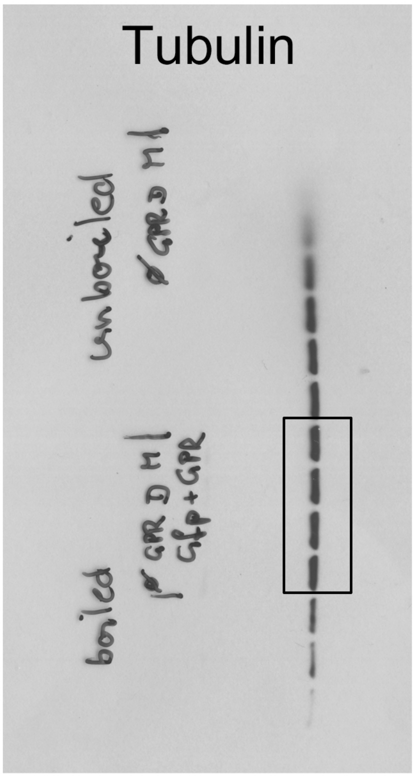

DENR & MCTS1

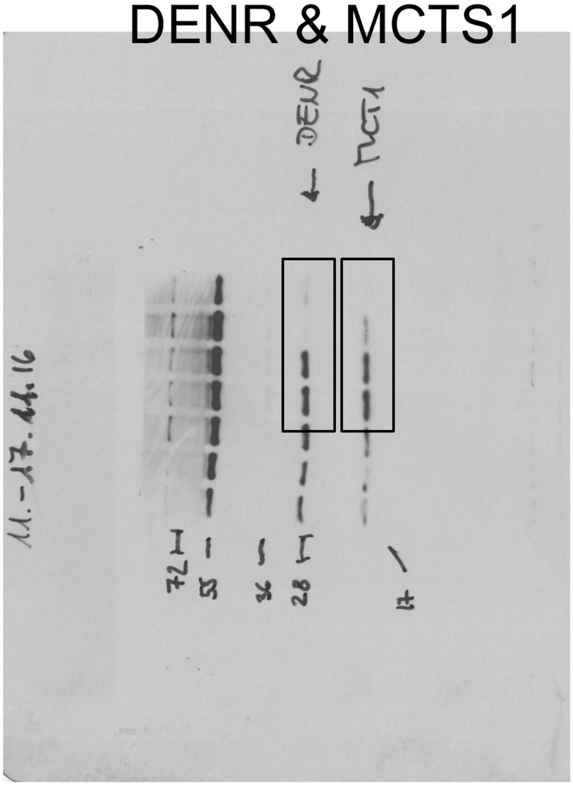

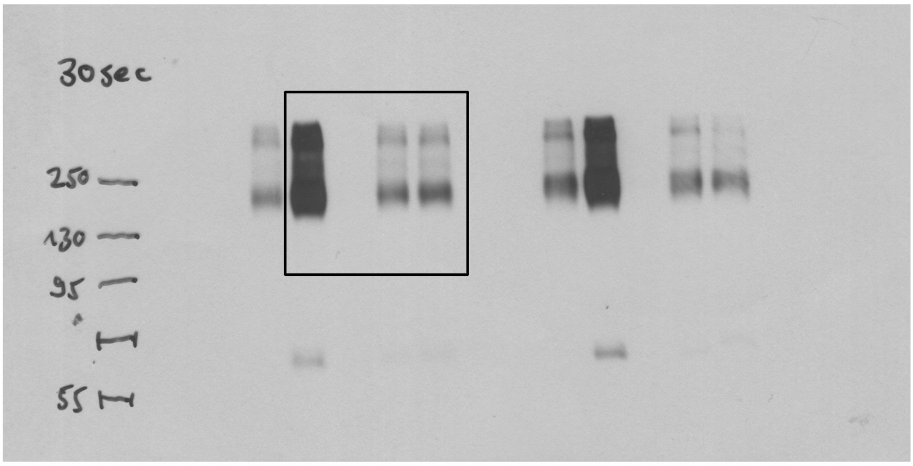

GPR37

Supplementary Figure Legends

Supplementary Figure S1: Support to Main Figure 1

(A-B) The drop in expression of the stuORF reporter upon DENR (A) or MCTS1 knockdown (B) is rescued by re-introducing expression of DENR or MCTS1, respectively, using overexpression (OE) constructs that have been mutated via synonymous codon changes to escape siRNA-mediated suppression.

(C-C') Ligatin (eIF2D) knockdown in HeLa cells does not affect expression of the stuORF reporter, perhaps due to relative stoichiometry of DENR, MCTS1 and eIF2D in HeLa cells [12]. (C) Unlike knockdown of DENR or MCTS1, knockdown of eIF2D does not cause a drop in expression of a stuORF containing luciferase reporter. (C') Immunoblot control for eIF2D knockdown efficiency.

Error Bars: Std. dev. ***student ttest $p \leq 0.001$.

Supplementary Figure S2: Support to Main Figure 2

(A) Non-normalized values for the luciferase assay in Main Figure 2A. Caveat: since the RLuc and FLuc reporters are on separate plasmids, these RLuc/FLuc ratios depend on the relative transfection of the two plasmids and hence are not robust and should be interpreted with caution.

(B-B') Multiple copies of a stuORF lead to strong dependence on DENR and MCTS1 for expression of the main ORF. (B) Schematic diagram showing the stuORF sequence that was cloned into the 5'UTR of a control reporter by

oligo cloning. By repeated rounds of oligo cloning, the sequence was inserted 1, 2, or 3 times in tandem. (B') Luciferase assay reveals that the down-regulation of translation upon DENR knockdown becomes stronger with higher stuORF copy numbers. Student ttest $^{2}p \leq 0.01$, $^{3}p \leq 0.001$ relative to ctrl RNAi.

(C-C') Dependence on DENR or MCTS1 for translation of the main ORF varies according to the length of the stuORF, with short stuORFs coding for only 1 amino acid imparting the strongest dependence. The experiment and results are similar to Main Figure 2C, except that here a stronger Kozak (gcgagtATGg) is used (compared to acaaaATGt in Main Figure 2C), and the sequence of the stuORF open reading frame is not repetitive, but a random complex sequence of amino acids. Student ttest $^{3}p \leq 0.001$ relative to ctrl RNAi.

Error Bars: Std. dev.

Supplementary Figure S3: Support to Main Figure 3

(A-A') Suppression of luciferase reporter activity upon DENR knockdown for stuORF containing 5'UTRs (A') is not accompanied by a drop in mRNA levels for these reporters (A), in agreement with an underlying translational impairment causing the suppression upon DENR knockdown. Student ttest $^{1}p \leq 0.05$, $^{2}p \leq 0.01$ relative to ctrl RNAi.

(B-B') The luciferase reporter bearing the DRD1 5'UTR, which includes an intron, is spliced in vivo when transfected into HeLa cells. (B) Schematic diagram of the DRD1 5'UTR luciferase reporter, showing the two pairs of

oligos used, one to detect splicing of the DRD1 5'UTR intron, and one to detect the renilla luciferase (RLuc) coding sequence as a normalization control. (B') Quantitative PCR detecting the spliced DRD1 5'UTR, normalized to the RLuc coding sequence, using as template either DRD1 reporter plasmid DNA, or cDNA from HeLa cells transfected with the DRD1 reporter. Error Bars: Std. dev. ***student ttest $p \leq 0.001$.

Supplementary Figure S4: Support to Main Figure 4

(A-B') Drop in GPR37 protein levels upon DENR or MCTS1 knockdown is not due to a drop in GPR37 mRNA levels (A) or protein stability (B-B'). (A) mRNA levels of the transfected GPR37 construct were quantified by Q-RT-PCR in HeLa cells treated with indicated siRNAs. GPR37 siRNA serves as a positive control. (B-B') GPR37 protein is very stable, and at least as stable upon DENR or MCTS1 knockdown as in the control condition. (B) Protein stability was assessed after 2 hours of cycloheximide (CHX) treatment (50µg/mL) to block synthesis of new protein. Protein levels for the unstable protein c-Myc were used as a positive control for the CHX treatment. (B') Quantification of GPR37 protein levels shows that it is very stable, dropping by circa 10% after 2 hours of CHX treatment, and protein levels do not drop more in the DENR or MCTS1 knockdown condition compared to the control condition.

(C) GPR37 mRNA levels in various cell lines, measured by quantitative RT-PCR, normalized to hRpL19.

(D) Knockdown of DENR or MCTS1 only mildly impairs proliferation of HeLa cells compared to a control knockdown. Cell counts were performed in the indicated days relative to siRNA transfection.

Error Bars: Std. dev. **student ttest $p \leq 0.01$.

Supplementary Figure S5: Uncropped immunoblots for Main Figure 1A.

Supplementary Figure S6: Uncropped immunoblots for Main Figure 4.
